# Performance Evaluation of UHF RFID Technologies for Real-Time Bus Recognition in the Taipei Bus Station

**DOI:** 10.3390/s130607797

**Published:** 2013-06-18

**Authors:** Chung-Ming Own, Da-Sheng Lee, Ti-Ho Wang, De-Jun Wang, Yu-Lun Ting

**Affiliations:** 1 Department of Computer and Communication Engineering, St. John's University, New Taipei City 25135, Taiwan; E-Mail: cmown@mail.sju.edu.tw; 2 Department of Energy and Refrigerating Air-Conditioning Engineering, National Taipei University of Technology, Taipei City 55106, Taiwan; 3 Department of Electronic Engineering, St. John's University, New Taipei City 25135, Taiwan; E-Mail: dhwang@mail.sju.edu.tw; 4 Wan Da Tong Enterprise Company, Taipei City 10351, Taiwan; E-Mails: derek@radium.com.tw (D.-J.W.); wodniw@radium.com.tw (Y.-L.T.)

**Keywords:** Taipei Bus Station, system platform, radio frequency identification (RFID), ultra-high frequency (UHF)

## Abstract

Transport stations such as airports, ports, and railways have adopted blocked-type pathway management to process and control travel systems in a one-directional manner. However, this excludes highway transportation where large buses have great variability and mobility; thus, an instant influx of numerous buses increases risks and complicates station management. Focusing on Taipei Bus Station, this study employed RFID technology to develop a system platform integrated with modern information technology that has numerous characteristics. This modern information technology comprised the following systems: ultra-high frequency (UHF) radio-frequency identification (RFID), ultrasound and license number identification, and backstage graphic controls. In conclusion, the system enabled management, bus companies, and passengers to experience the national bus station's new generation technology, which provides diverse information and synchronization functions. Furthermore, this technology reached a new milestone in the energy-saving and efficiency-increasing performance of Taiwan's buses.

## 1. Introduction

Since the Industrial Revolution in the eighteenth century, the enhancement of industrial technology, continuous development of technological civilization, and the exceeding industrial value over agricultural value, has increased job opportunities and enhanced material well-being in metropolitan regions. Consequently, people from rural areas continue to migrate to cities that are expanding because of urbanization, resulting in the continuous development of large metropolitan areas. Although resources for urban lands are limited, the improvement of industrial technology still promotes the rapid development of motor vehicle companies, and the government's transport policy still focuses on constructing highly efficient highways at low cost to enable rapid and convenient travel [1]. These factors enhance suburbanization and cause cities to extend into suburban areas, displaying linear, radial, and wave-like developmental patterns. This development also results in housing-job imbalances, increased transport construction costs, public facility and resource wastage, greater governmental financial obligations, increased public spending, and serious environmental pollution [2–4]. The government's unplanned developmental strategies that focus on highway development and public demand have greatly influenced the efficient use of land resources.

Taiwan is located off the southeastern coast of China, at the western edge of the Pacific Ocean, between Japan and the Philippines. Taipei City is the most modern and the largest city in Taiwan. In Taipei, there are more than 3 million people, but more half of the 3 million people who are from southern or central Taiwan work and live in this city. Because of spatial limitations and population density, the roads of the Taipei Metropolitan Area are restricted by the norms of urban planning and limited land resources. Consequently, existing urban infrastructures become inadequate, transport constructions fall behind schedule, and road cannot be effectively integrated with land utilization, causing chaotic traffic in urban areas, inefficient development of public facilities, accelerated environmental deterioration, and increased social costs. Before Taipei Bus Station began operations, the bus stations in central Taipei that served long-distance highway travel were located around Taipei Main Station. Several long-distance bus companies have established bus stations in New Taipei City, specifically the Banqiao and Sanzhong districts; however, most Taipei citizens prefer to use the bus stations around Taipei Main Station when traveling on long-distance buses (hereafter referred to as buses). Citizens returning from other counties and cities often travel to Taipei Main Station and then transfer to other transportation methods to travel from downtown (Taipei) to neighboring areas. Consequently, traffic has become chaotic in the major locations around Taipei Main Station, causing numerous citizens to make unnecessary trips to and from Taipei and neighboring cities. Because bus stations are situated at Taipei Main Station, buses must detour extended lengths before entering highway interchanges. This not only extends travel time, but also reduces operational efficiency and increases operation costs. In addition, city traffic during peak hours lengthens transportation times of buses, further affecting to surround traffic condition around bus stations. Therefore, to meet the goals of Taipei's development and promote public transportation policy, the government began the overall planning and construction of a center for long-distance bus travel services in 2009, that is, the Taipei Bus Station.

Comparing to other bus stations, the Taipei Bus Station employs the UHF Radio Frequency Identification (RFID) detecting technology as the core solution. RFID is an electronic tagging technology that allows objects, places, or persons to be automatically identified from a distance without a direct line-of-sight, using an electromagnetic challenge/response exchange [5,6]. RFID offers a possible alternative to barcodes, which has emerged as a key technology for a wide-range usage in the supply chain, retail stores and asset management [7]. In our study, RFID positioning technology integrates and transmits bus companies' schedule information, effectively and flexibly dispatches buses, increases bus usage, and consequently enhances operational efficiency. Thus, focusing on Taipei Bus Station, we employed RFID technology to develop a system platform integrated with modern RFID information technology. This system can automatically provide the bus status and schedule to management and bus companies via various devices for information detection, collection and professional filed models for judgments.

The rest of this paper is organized as follows: we discuss related work in Section 2. Section 3 defines the system framework for the Taipei Bus Station. The system performance and analysis are reported in Section 4. Finally, Section 5 concludes the paper.

## 2. Literature Review

### 2.1. Transfer Station Definition and Introduction

**Figure 1.**
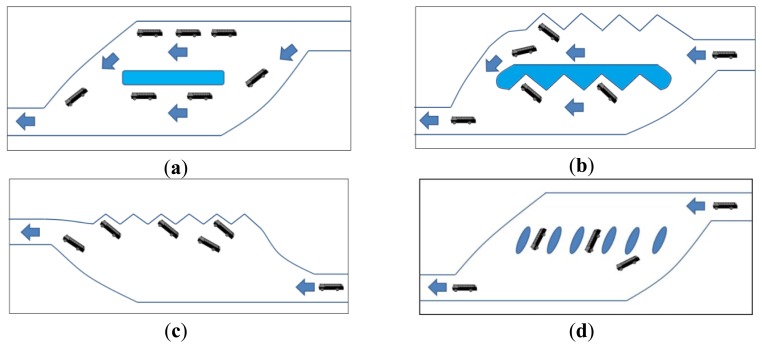
Bus station platform layout types, (**a**) the straight-line layout; (**b**) the zigzag layout; (**c**) the obtuse-angle layout; (**d**) the leaving-type layout.

Public transport transit centers have been operating in the main cities of Europe, the United States, and Japan for many years, effectively improving public transportation developments and road traffic congestion. By expediting and establishing a public transport transit center that are supported by the central government and local industries, Taiwan aims to effectively resolve the current problems related to road traffic congestion in major cities, and then implement policies regarding the “Mass Transportation Development Project.” Furthermore, scholars of extant literature have been varying definitions for bus stations. Based on the Transportation Research Board (TRB)'s definition, the various platform types in bus stations can be classified into the four categories listed in Figure 1[8]. Their features are listed as follows:

- The straight-line layout: this layout is for the inefficient land use, the short stopping durations, and suitable for roadside bus stops.
- The zigzag layout: this layout is for the separate entry and exit platforms for large buses, and suitable for bus transit centers.
- The obtuse-angle layout: this layout is for the long stopping duration, and suitable for intercity. The effective area facilitates numerous platforms. This layout reserves the space for the large buses to exit.
- The leaving-type layout: this layout is for the independently used platform, and suitable for bus transit centers. Besides, the land use is restricted.

**Figure 2.**
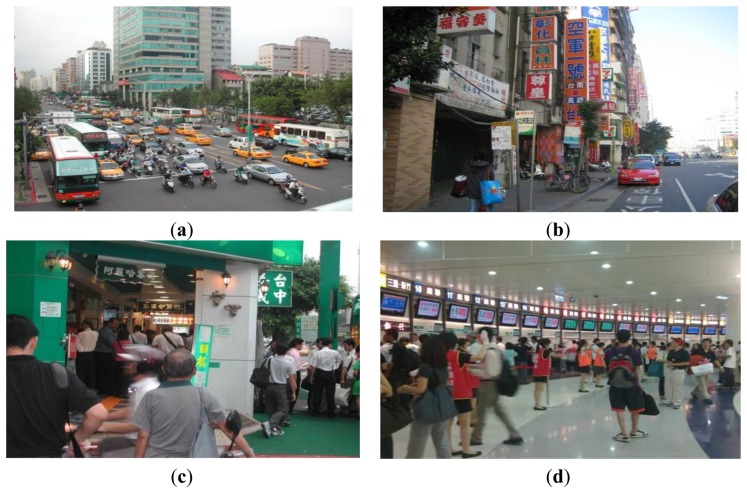
(**a**) Traffic on Chengde Road; (**b**) roadside bus stop signs. Each bus company has a different sign; (**c**) Crowds of boarding passengers on Chengde Road; and (**d**) Taipei Bus Station ticket counters.

Before 2009, Taipei adopted a straight-line platform layout; this allowed buses to stop around Taipei Railway Station and along one side of Chengde Road. However, the varying business stores and signs gave the surrounding area a disorganized and confusing appearance [Figure 2(a-c)]. In addition, the current design of Taipei Bus Station is based on the obtuse-angle platform layout. Various bus companies employed the ballot method to select platforms at different levels. Furthermore, the bus station system controls the bus stopping time of each transport company. Improvements to the passenger waiting areas are shown in Figure 2(d).

**Figure 3.**
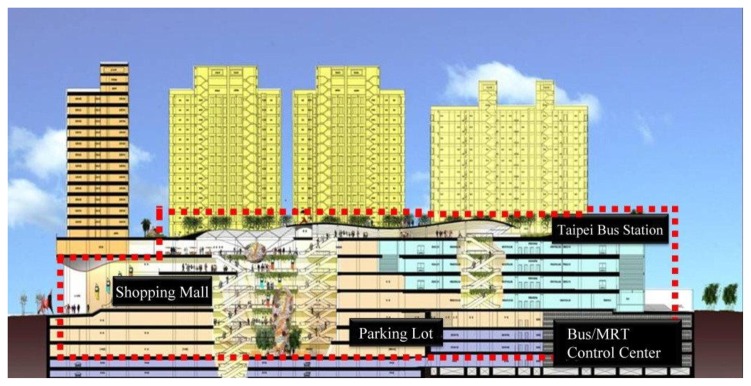
Taipei Bus Station floor plan.

In Taipei, 36 national bus companies have been approved by the Directorate General of Highways (MOTC) serving more than 170 routes, 99 of which depart from or arrive in Taipei City. Taipei Bus Station has 15 bus companies serving 65 routes and an average of 2,289 and 2,427 buses scheduled per day for normal and peak days, respectively [9]. Taipei Bus Station is the transport hub for the Taipei Metropolitan Area. Served by five modes of transport [the Taiwan Railway (TRA), TaiwanHigh-speed Rail (THSR), Mass Rapid Transit (MRT) system, Taoyuan International Airport MRT (Taoyuan MRT), and highway buses], this transfer station is located at the center of Western Taipei City and north Taipei Main Station, and near Civic Boulevard, Chengde Road, and Huayin Street. The Taipei Bus Station facility is a three-dimensional building covering 28,086 m^2^ and four floors. The first floor contains ticket counters, and the second to fourth floors are the waiting areas with central-island platforms with 16 platforms (*i.e.*, 48 total parking platforms) on each floor. In addition, the station has one entrance and two exits, and the lanes are unidirectional. The buses on the ground floor lane travel clockwise. One entrance on Chengde Road serves as a bus entrance and exit, with another exit to Civic Boulevard provided on the third floor, enabling buses to connect to Huanhe Highway via the expressway, and then rapidly exit the city via National Freeway No. 1. This avoids the traffic interference caused by buses, enhances buses' transit rates, and reduces buses' consumption of oil when idle. Figure 3 shows the floor plan of Taipei Bus Station.

### 2.2. Introducing Taipei Bus Station

During the planning of Taipei Bus Station, the following design principles were considered [9]:

1. Avoid interwoven routes of travel: To increase passenger safety in the bus station and enhance the operational flow of buses, separation of the movement paths for people and buses was adopted as the principle.
2. Convenient transfers: The primary function of the bus station was to enhance the convenience of transferring between various transport methods.
3. Physically separate the platforms and waiting areas: To prevent the fumes emitted by buses affecting the passengers, the waiting areas and the platforms were physically separated, thereby reducing the effects that gases emitted by the buses have on the passengers.

### 2.3. The Radio Frequency Identification System (RFID)

RFID is a general term that is used to describe a system that transmits the identity with the unique serial number wirelessly [5,6]. This is sometimes referred to as contact-less technology and a typical RFID system is made up of three components: tags, readers and the host computer system. An RFID tag is a tiny radio device that is also referred to as a transponder, smart tag, smart label or radio barcode. The tag comprises of a simple silicon microchip attached to a small flat aerial and mounted on a substrate. The finished tag can be attached to an object, typically an item, box or pallet and read remotely to ascertain its identity, position or state. Battery-powered tags may operate at hundreds of meters. Unlike a bar code, the tag does not necessarily need to be within the line of sight to the reader, and may be embedded in the tracked object. Besides, the reader, sometimes called an interrogator or scanner, sends and receives RFID data to and from the tag via antennas. A reader may have multiple antennas that are responsible for sending and receiving radio waves.

There is a total of four different RFID frequency bands (frequencies) that used across the globe. Like tuning in to your favorite radio station, RFID tags and readers must be tuned into the same frequency to enable communications. RFID systems can use a variety of frequencies to communicate, but because radio waves work and act differently at different frequencies, a frequency for a specific RFID system is often dependent on its application. Ultra High Frequency (UHF) RFID systems (850 MHz to 950 MHz and 2.4 GHz to 2.5 GHz) offer transmission ranges of more than 90 feet, although wavelengths in the 2.4 GHz range are absorbed by water, which includes the human body, and therefore has limitations.

## 3. System Framework

The purpose of intelligent RFID in transit systems was to employ modern information technology to provide a new-generation bus station. The scope of RFID encompasses collecting information regarding bus schedules, managing station affairs, profit sharing and liquidation, controlling active buses and routes within the station, license plate identification, image monitoring, adjusting platform settings, warning of bus abnormalities, and even the provision of information regarding ticket purchasing, public service websites, and a public information station. We endeavor to platform transfer efficiency in the bus station and increase the standards, diversity, and convenience of the station's services through the electronic, communication, information, and transport management technologies capabilities of RFID.

## 3.1. The RFID Management System

To achieve intelligent management, the system employs an automatic ultra-high frequency (UHF) RFID tag detector as the primary technology for license plate identification and station management. RFID technology has advanced to signify over the past few decades. Rapid advances in microelectronic transceivers have reduced the size and costs of HF and UHF RFID infrastructure, permitting the longer reading ranges and faster reading rates than ever before [10,11]. Thus, UHF RFID technology is the core solution to our system.

**Figure 4.**
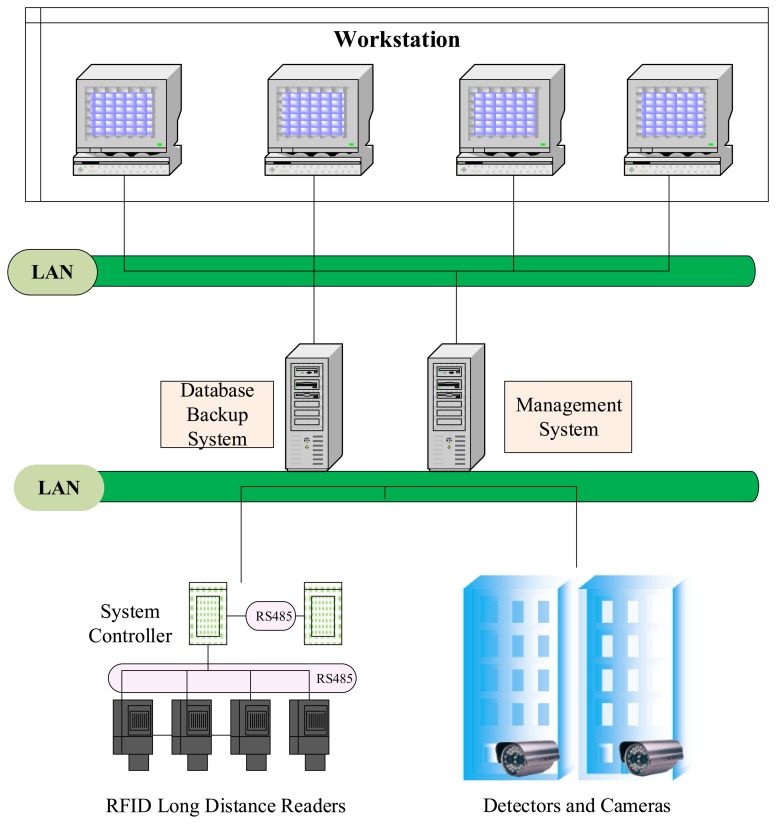
Framework of the RFID management system.

In addition, the graphic control system allows administrators to fully control the situation regarding active buses within the station and to monitor abnormal conditions. The RFID technology is used to identify active buses. Reader devices are installed in relevant positions within the bus station to identify each bus as it enters and exits and the positions of the buses in the station. Furthermore, these readers are synchronized with detectors and cameras installed at the entrance, exits, and platforms to ensure license plate numbers and verify passage and transit. Bus positions within the station are displayed as simple icons in real time on the monitors of the control center. The framework of the RFID management system is shown in Figure 4.

## 3.2. The UHF RFID Identification System for Bus Lanes and Platforms

Considering the current situations and criteria of the roads around the bus station, the entrance into the station is located on Chengde Road. The route of travel initially begins at the entrance to the station at Chengde Road, connecting to a ramp leading to the second floor. Proceeding clockwise along the ramp leads to the entrance to the second floor platforms. The ramp leading to the third floor is located on the east side of the second floor. Proceeding clockwise along this ramp leads to the third floor, which has a layout similar to that of the second floor. To the east side of the third floor is the ramp leading the fourth floor. For leaving the station, exits are provided on the ground floor onto Chengde Road, and on the third-floor onto Civic Boulevard.

**Figure 5.**
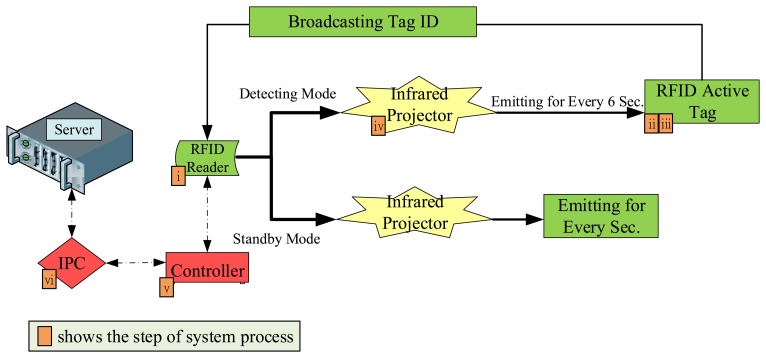
Function of the RFID reader regarding the lanes and platform.

The bus station is a closed environment. Because buses intertwine occasionally, internal positioning technologies are somewhat difficult to provide to the station system. Current systems apply automatic UHF RFID standards where the primary frequency is 433 MHz. Because a UHF reader device can read further, the system added a technology that employed infrared-activated RFID tag. The overall process is shown in Figure 5.

(i) With an interval of 4 seconds, the RFID reader controls the infrared projector, which emits infrared signals to the RFID tag on the bus. The information packets include the RFID reader's serial number. Infrared projectors in different locations have varying power (5 W to 9 W) with a maximum projection distance of 30 m.
(ii) When the RFID tag on the bus receives the signals, a device reads the serial number and adjusts the frequency according to the reader, which ranges between 430 MHz and 438 MHz.
(iii) The RFID tag on the bus transfers packets containing the card number and tag's battery information to the reader.
(iv) After receiving the signal, the RFID reader sends confirmation packets to the bus every 6 seconds to determine whether the bus has left the platform or lane.
(v) Because the information about the system is constantly active, every four RFID readers are connected to one controller, which is used for data aggregation.
(vi) Each industry PC (IPC) is connected to three controllers, the information exchanged between the controllers is transmitted through a RS-232. The IPC then stores the signals over Ethernet in the back-end Oracle database server.

**Figure 6.**
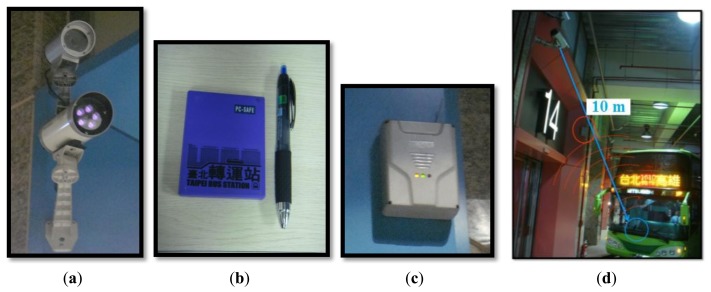
RFID reader equipment on the lanes and platforms; (**a**) is the infrared projector; (**b**) is the automatic UHF RFID tag on a bus; (**c**) is the UHF RFID reader; and (**d**) is a schematic diagram showing the RFID signal between the platform and the bus.

Figure 6 shows the equipment mentioned above in a schematic diagram. Figure 6(a) is an infrared projector; (b) is the automatic UHF RFID tag on a bus; (c) is the UHF RFID reader; and (d) is a schematic diagram of the read process between the bus and the platform. The reading distance is restricted to about 10 m.

## 3.3. The UHF RFID Active Tag

**Figure 7.**
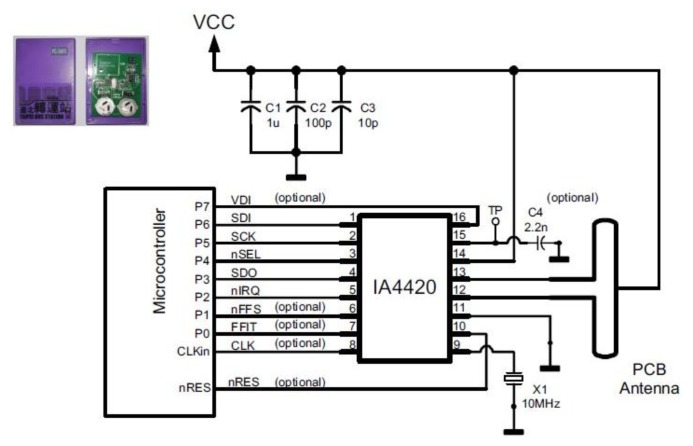
The functional block diagram of the UHF RFID active tag.

The active tag on the bus is combined by the infrared receiver, MSP430F112 chip and the IA4420 chip as the transmitter/transceiver at the 315, 433, 868 and 915 MHz ISM bands. The Programmable TX frequency is the deviation from 15 to 240 KHz, and the Programmable RX baseband bandwidth is from 67 to 400 kHz. When the tag waked up by the Infrared signal, the tag would encode the signal number and switch to the pre-defined frequency by the number. Figure 7 is the functional block diagram of the UHF RFID active tag.

## 3.4. System for Ultrasound and Vehicle Plate Identification

**Figure 8.**
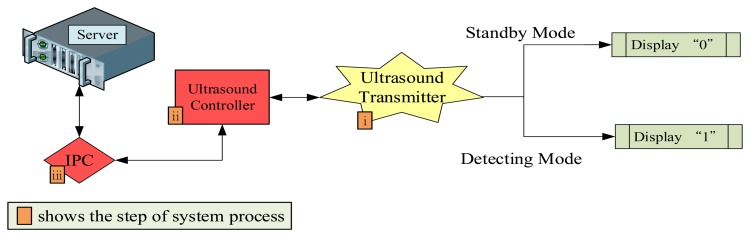
Process of ultrasound and vehicle plate identification.

system for ultrasound and vehicle plate identification was installed in the bus station as an auxiliary system to the RFID system. This auxiliary system prevents unknown buses entering the station and the loss of bus tag functions, which can cause the un-identification problems. The system process is as follows:

The overall identification process was as follows (Figure 8):

(i) Each ultrasound transmitter had an audio frequency of 40 KHz and transmitted the detected ultrasounds 4 times every second, this is the standby mode. During the standby mode, the displayed status is “0.” When the ultrasound detects a vehicle in front, the display on the ultrasound transmitter becomes “1,” and the system changes into detecting mode, the transmitting time is double. Subsequently the transmitter sends the signals to the buffer zone of the ultrasound controller.
(ii) Each controller is connected to four ultrasound transmitters, and compiles and transmits received signals to the IPC, which is connected to three ultrasound controllers.
(iii) Similar to the RFID data process, the IPC and ultrasound controller are based on a RS-232 connection. They compile and send the received signal through the power-line network to the back-end Oracle database server.
(iv) Cameras for the vehicle license plate identification system were installed at the entrances and exits. The identification system conducts a search for buses not registered on the system, compares information from the ultrasound controllers, and identifies buses not registered or with non-functional RFID tags.

**Figure 9.**
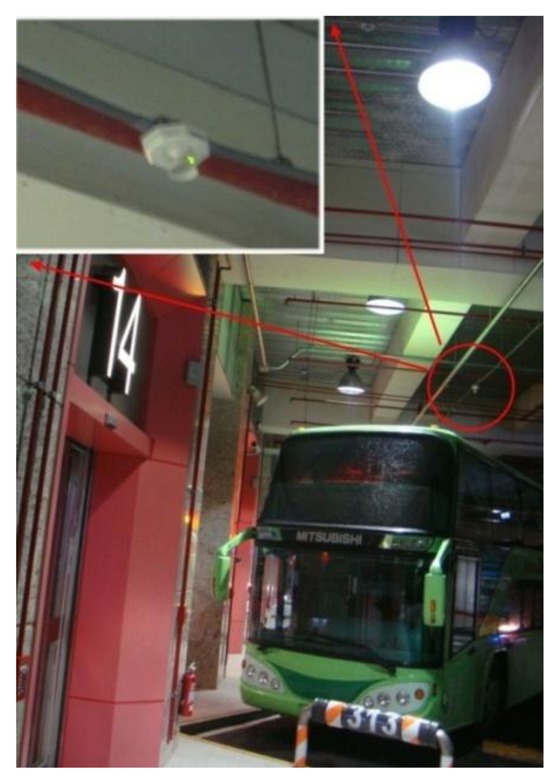
The actual ultrasound transmitter is located approximately 1m from the buses.

**Figure 10.**
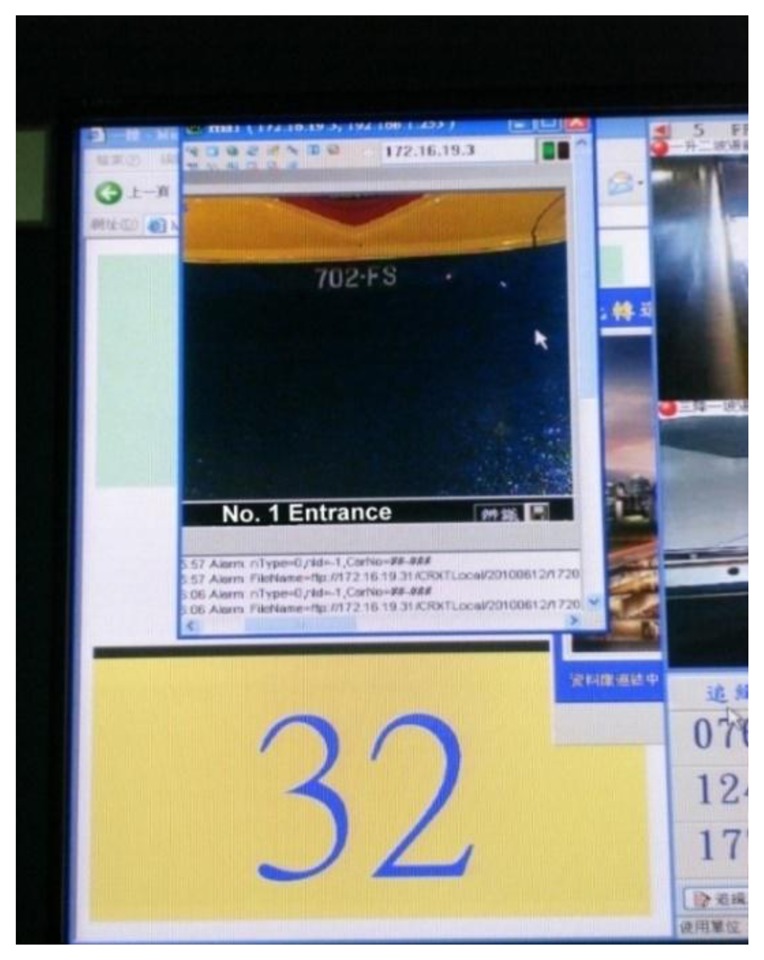
Vehicle license plate identification system.

Figures 9 and 10 show the ultrasound transmitter and license plate identification system of the station.

## 3.5. Backstage Graphic Control System

**Figure 11.**
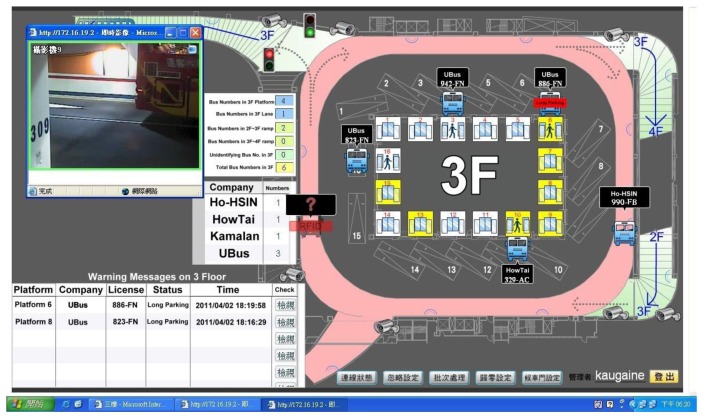
Interior images of the bus station captured by the graphic control system.

Taipei Bus Station adopted a station structure comprising of numerous floors. Adopting traditional operation approaches to manage the bus station results in excessive human resource costs for the station to operate normally. After integrating a graphic control system containing RFID and electronic maps in the bus station, the system was used to convert information from RFID to images, which was then displayed by the graphic control system. Consequently, only a few management employees are required and conditions in various areas of the station could be clearly and rapidly perceived. Figure 11 shows actual images of the station captured by the system.

## 4. System Performance and Analysis

### 4.1. System Performance

Currently, the national bus stations in Taipei comprise Taipei Bus Station, Taipei City Hall Bus Station, and Banqiao Bus Station. Measuring the distance between each bus station and the administrative center in each county and city district, the bus station with the shortest distance from the center for that district is considered the service provider. The jurisdictions of each bus station are tabulated as follows:

The bus management systems employed by the three bus stations are listed as follows: The system adopted at the Taipei Bus Station is the RFID management system, and both the Taipei City Hall Bus Station and the Banqiao Bus Station employed the license plate identification system. Considering the number of platforms available during peak hours, the formula (*BF_i_*) used in this study to calculate the station supply capacity was:

$${\text{BF}}_{i}=B_{i}\times {\text{OT}}_{i},$$

where the number of platforms *B_i_* in the station is multiplied by the operation time *OT_i_* and the value obtained represents the available number of platforms during peak hours. Because the operation times of each bus station and the standard times when travelers accumulate to differ, we based ou calculations on the peak times when the buses are operating. Between 5 p.m. and 8 p.m., the maximum number of platforms available at Taipei Bus Station, Taipei City Hall Bus Station, and Banqiao Bus Station were 102, 48, and 33, respectively [12–14]. Aside from accurately planning operations in advance for the bus station, the system controlling the entire station also plays an extremely crucia role. To compare the system performance of each bus station, we generated measurement data of the following: the number of buses stopping at peak hours divided by the number of platforms at peak hours. Table 1 is the listed of districts served by each bus station.

Furthermore, according to descriptions [12–14], Table 2 is the collection of station information.

**Figure 12.**
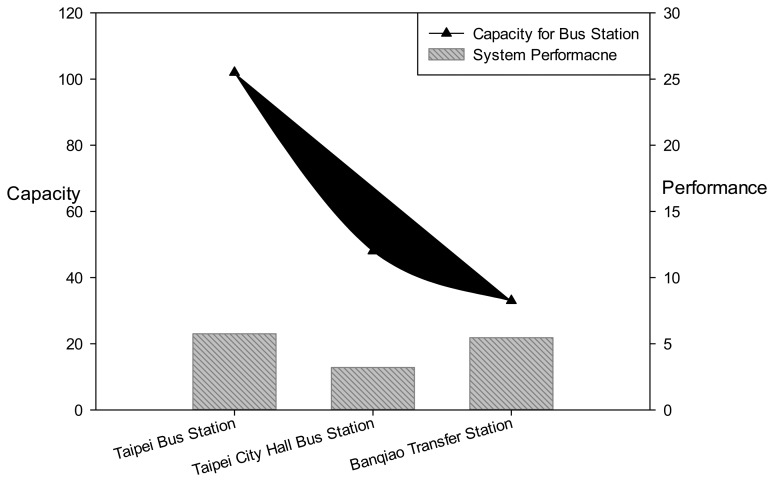
The efficiency comparison for the three bus stations.

Figure 12 comparative graph showing the efficiency of the bus stations' system performance. The system performance (*sp*) is calculated by the derived numbers during peak hours:

$$\text{sp}=\frac{\text{Number of buses}}{\text{Number of platforms}}.$$

According to Table 3, the system performance of the Taipei Bus Station, Taipei City Hall Bus Station, and Banqiao Bus Station was 5.75, 3.20, and 5.45, respectively. These results show that each platform in Taipei Bus Station can provide services to more people, which suggests that the bus system employed at Taipei Bus Station is superior.

### 4.2. System Performance Analysis

The Taipei Bus Station operates 24 h a day, and a daily average of approximately 5,000, 6,300, and 520 buses enter and exit the station during standard, peak days, and peak hours, respectively. When the maximums of 89 buses are present in the station, the buses are still operated systematically. Besides, accordingly to [15], there is the face to face survey in Taipei Bus Station between July and August 2011. The travelers' questionnaires were issued, were issued by convenience sampling of 450 questionnaires and collected 436 questionnaires. The overall response rate was 96%. The travelers' willingness study revealed that there are six items over the above-average satisfaction in the Taipei Bus Station, included the clean station, the comfortable waiting area, quickly ticket purchase, real-time bus information, on-time bus schedule and precise bus routes, and smooth traffic foot flow. The half items are under the proposed system control. Thus, the system contribution is irreplaceable. The stability of the RFID management system is a crucial factor, enabling it to detect events rapidly and accurately for efficient management. The system performance is explained below.

- Intelligent station management
  (A) Monitor bus abnormalities: When buses have stopped or are circling within the station without cause, the system automatically detects “incidents” or “congestion” and activates RFID positioning for interpretation.
  (B) Monitor number of buses: Instantly displays the number of buses on the platforms, lanes, and ramps in the station, and immediately calculates the number of company buses on each floor.
  (C) Identify buses on the platforms: Buses parking incorrectly or stopping for too long on the platforms are identified; the system automatically captures images and transmits warning messages.
  (D) Interactive and instant monitoring: If bus companies violate the station regulations, the system immediately transmits images to the offending company's station management computer; if the management of that bus company does not process and report the situation instantly, the system's passenger boarding operations cannot be initiated *(i.e.*, platform doors and broadcasting boarding announcements cannot be activated).
- Enhancement of the management performance of bus companies
  (A) Dispatch precise number of buses: Provides locations for incoming buses and lanes for buses within the station; station management can then prepare to board operations in advance. Based on data regarding bus operation times provided by the system, bus companies can continuously optimize the scheduling mode, increase the platform turnover rate, and enhance the platform performance.
  (B) Automatic boarding operation: According to the routes and license plate numbers registered by the companies, the system identifies the buses, reminds station staff when buses enter the station, confirms identification when buses stop at the platforms, and activate a series of boarding operations. The station staff can then conduct ticket inspections and facilitate passenger boarding.
- Energy-saving performances
  (A) After buses are parked at the platform and their identification confirmed, power is provided to the platform doors and ventilation systems; when buses leave the platforms, the power is switched off.
  (B) Vortex-type extractor fans installed on the lanes are activated when the system detects a certain number of buses in the station (concentrations of polluted air and gas in the station are calculated according to the amount of gas released by the buses), that is, the system activates each floors' extraction facilities accordingly to enhance and ensure air quality in the station.
- Additional performance factors
  (A) Manage bus identification: Each bus has a unique RFID tag identifier. If buses exhibit abnormalities or are parked at the wrong platform, the system can instantly notify and trace the buses. The compiled information regarding the identity of each company's bus is used by highway project management units for bus control.
  (B) Tracing incidents: Buses involved in an incident are identified according to time and location.

## 5. Conclusions

Transport stations such as airports, ports, and railways have adopted blocked-type management to maintain unidirectional air routes and tracks, excluding highway transport, where large bus transport vehicles have considerable variability and mobility. Therefore, an instant influx of numerous buses complicates and increases risks during station management. In this study, the primary characteristic of the management system for Taipei Main Station was its construction on an integrated platform. This platform automatically provided the bus status and schedules to management and bus companies via various devices for information detection and collection and professional field models for judgments. Management immediately perceives bus movements and abnormalities in the station. Thus, bus companies can provide information of bus schedules from the integrated platform to general passengers through the platform broadcasting and multimedia billboard system. Consequently, the intelligent management and operation system for Taipei Bus Station reduces labor costs and allows users to control information regarding bus schedules.

RFID positioning technology integrates and transmits bus companies' schedule information, effectively and flexibly dispatches buses, increases bus usage, and consequently enhances operational efficiency. The technology allowed management, bus companies, and passengers to experience the national bus station's new-generation technology, which provides diverse information and synchronizes functions. In conclusion, this technology has reached a new energy-saving and efficiency-increasing milestone for Taiwan's buses.

## Figures and Tables

**Table 1.** Districts served by each bus station.

**Taipei Bus Station**		**Taipei City Hall Bus Station**		**Banqiao Bus Station**	
Zhongzheng	Yonghe	Songshan	Shiding	Xinzhuang	Taishan
Wanhua	Sanzhong	Xinyi	Ruifang	Shulin	Banqiao
Datong	Luzhou	Nankang	Pinglin	Tucheng	Linkou
Zhongshan	Sanzhi	Neihu	Pingxi	Yingge	Wugu
Da′an	Tamsui	Wenshan	Shuangxi	Zhonghe	
Shilin	Bali	Xindian	Gongliao	Sanxia	
Beitou	Shimeng				

**Table 2.** Bus station information [12–14].

**Platforms and Temporary Parking Facilities**	**Taipei Bus Station**	**Taipei City Hall Bus Station**	**Banqiao Bus Station**
Boarding/alighting platforms	34 platforms	16 platforms	10 platforms
Temporary parking platform	16 platforms	7 platforms	1 platform
Passenger waiting areas	1083 m^2^ × 3	1615 m^2^	745 m^2^
Platforms available during peak hours	102	48	33
Number of buses during peak hours	Approx. 587	Approx. 154	Approx. 180
Passenger transport bus management system	UHF RFID	License plate identification	License plate identification
